# Replication of genetic associations of chemotherapy-related cardiotoxicity in the adjuvant NSABP B-31 clinical trial

**DOI:** 10.3389/fonc.2023.1139347

**Published:** 2023-05-25

**Authors:** Pooja P. Advani, Kathryn J. Ruddy, Joerg Herrmann, Jordan C. Ray, Emily C. Craver, Greg Yothers, Reena S. Cecchini, Corey Lipchik, Huichen Feng, Priya Rastogi, Eleftherios P. Mamounas, Sandra M. Swain, Charles E. Geyer, Norman Wolmark, Soonmyung Paik, Katherine L. Pogue-Geile, Gerardo Colon-Otero, Edith A. Perez, Nadine Norton

**Affiliations:** ^1^ Department of Hematology and Oncology, Mayo Clinic, Jacksonville, FL, United States; ^2^ Department of Oncology, Mayo Clinic, Rochester, MN, United States; ^3^ Department of Cardiovascular Diseases, Mayo Clinic, Rochester, MN, United States; ^4^ Department of Cardiovascular Diseases, Mayo Clinic, Jacksonville, FL, United States; ^5^ Department of Health Sciences Research, Mayo Clinic, Jacksonville, FL, United States; ^6^ NRG Oncology Statistics and Data Management Center, Pittsburgh, PA, United States; ^7^ Department of Biostatistics, The University of Pittsburgh, Pittsburgh, PA, United States; ^8^ NRG Oncology/NSABP Foundation, Pittsburgh, PA, United States; ^9^ UPMC Hillman Cancer Center, University of Pittsburgh School of Medicine, Pittsburgh, PA, United States; ^10^ Department of Surgical Oncology, Orlando Health Cancer Institute, Orlando, FL, United States; ^11^ Department of Surgical Oncology, Georgetown Lombardi Comprehensive Cancer Center, Washington, DC, United States; ^12^ Department of Cancer Biology, Mayo Clinic, Jacksonville, FL, United States

**Keywords:** anthracycline, doxorubicin, trastuzumab, breast cancer, cardiomyopathy, cardiotoxicity, TRPC6

## Abstract

**Background:**

The cardiotoxic effects of doxorubicin, trastuzumab, and other anticancer agents are well known, but molecular genetic testing is lacking for the early identification of patients at risk for therapy-related cardiac toxicity.

**Methods:**

Using the Agena Bioscience MassARRAY system, we genotyped *TRPC6* rs77679196, *BRINP1* rs62568637, *LDB2* rs55756123, *RAB22A* rs707557, intergenic rs4305714, *LINC01060* rs7698718, and *CBR3* rs1056892 (V244M) (previously associated with either doxorubicin or trastuzumab-related cardiotoxicity in the NCCTG N9831 trial of anthracycline-based chemotherapy ± trastuzumab) in 993 patients with HER2+ early breast cancer from the NSABP B-31 trial of adjuvant anthracycline-based chemotherapy ± trastuzumab. Association analyses were performed with outcomes of congestive heart failure (*N* = 29) and maximum decline in left ventricular ejection fraction (LVEF) using logistic and linear regression models, respectively, under an additive model with age, baseline LVEF, and previous use of hypertensive medications as covariates.

**Results:**

Associations of maximum decline in LVEF in the NCCTG N9831 patients did not replicate in the NSABP B-31 patients. However, *TRPC6* rs77679196 and *CBR3* rs1056892 were significantly associated with congestive heart failure, *p* < 0.05, with stronger associations observed in patients treated with chemotherapy only (no trastuzumab) or in the combined analysis of all patients relative to those patients treated with chemotherapy + trastuzumab.

**Conclusions:**

*TRPC6* rs77679196 and *CBR3* rs1056892 (V244M) are associated with doxorubicin-induced cardiac events in both NCCTG N9831 and NSABP B-31. Other variants previously associated with trastuzumab-related decline in LVEF failed to replicate between these studies.

## 1 Introduction

Joint analyses of the landmark NSABP B-31 and NCCTG N9831 clinical trials showed significantly improved disease-free survival (DFS) and overall survival (OS) with adjuvant chemotherapy (doxorubicin, cyclophosphamide, and paclitaxel) and trastuzumab in patients with HER2+ early breast cancer (1). In these clinical trials, cardiotoxicity was the major adverse event of trastuzumab in the form of left ventricular ejection fraction (LVEF) decline (often asymptomatic) and, less frequently, congestive heart failure (CHF). However, trastuzumab in combination with chemotherapy carries a favorable benefit-to-risk ratio and continues to be the standard of care.

Separate long-term cardiac safety analyses of the NSABP B-31 (2) and NCCTG N9831 (3) trials both showed a higher cumulative incidence of cardiac events (defined as definite or probable cardiac death or symptomatic congestive heart failure) in patients treated with chemotherapy plus trastuzumab (4.0% and 3.0%, respectively) compared to chemotherapy alone (1.3% and 0.6%). After stopping trastuzumab, most patients who experienced cardiac dysfunction recovered LVEF to within normal limits, although some decline from baseline often persisted. In both of these studies, the incidence of late-onset cardiac events was very low, suggesting that the main cause of CHF is the result of anthracycline exposure, which has long been known to cause CHF (4, 5). However, a large retrospective study based on SEER codes showed a significantly higher frequency of heart failure/cardiomyopathy in breast cancer patients treated with trastuzumab alone or anthracycline plus trastuzumab (HR = 4.12 and 7.19, respectively) than with chemotherapy alone (HR = 1.40) (6).

Both the NSABP B-31 and NCCTG N9831 clinical trials identified older age, lower baseline LVEF, and use of hypertensive medications as risk factors for CHF, but prediction of those patients who progress from asymptomatic decline in LVEF to CHF remains poor. In an attempt to identify additional factors that may improve risk prediction, a genome-wide association study (GWAS) of the maximum decline in LVEF in DNA specimens from the NCCTG N9831 study, controlling for age, baseline LVEF, and use of hypertensive medications, was previously performed (7). The N9831 GWAS study identified genetic associations of the maximum decline in LVEF at six novel loci (*LDB2*, *BRINP1*, chr6 intergenic, *RAB22A*, *TRPC6*, and *LINC01060*) in the subset of patients who were treated with chemotherapy plus trastuzumab (*p* < 1 × 10^−5^) and in the same patient group, with the *CBR3* V244M variant (*p* < 0.05), which has previously been associated with anthracycline-induced cardiomyopathy and heart failure, summarized in (8). As an attempt to validate our previous data, in the current study, we examined the same variants in DNA specimens from the NSABP B-31 trial, assessing their associations with the maximum decline in LVEF and with incidence of CHF.

## 2 Methods

### 2.1 Patient population

Patients in the NSABP B-31 clinical trial gave written, informed consent for participation and the studies for genetic associations were approved by the institutional review board at the Mayo Clinic. Although the designs of the NSABP B-31 and N9831 trials were fairly similar, there were some differences that may be relevant for the purpose of comparison of cardiotoxicity. In the NSABP B-31 trial, patients were randomized to two treatment arms. In Arm 1, patients received doxorubicin, cyclophosphamide every 3 weeks for four cycles, then paclitaxel alone every 3 weeks for four cycles; in Arm 2, patients received the same sequential chemotherapy regimen with the initiation of a year of trastuzumab concurrent with the paclitaxel administration. Patients received serial multigated acquisition scans (MUGA) at baseline, 3, 6, and 9 months after registration, and after completion of chemotherapy, and some women consented for a follow-up evaluation. Cardiac events were defined as either symptomatic CHF confirmed by MUGA or echocardiogram or probable/definite cardiac death (CD) as reviewed by an external cardiac advisory panel consisting of three cardiologists. A majority decision of the cardiac advisory panel determined whether the protocol criteria for a CE were met.

In the NCCTG N9831 trial, patients were randomized to either Arm A (doxorubicin, cyclophosphamide followed by paclitaxel given weekly ×12) alone, Arm B (doxorubicin, cyclophosphamide followed by weekly paclitaxel followed by trastuzumab given for 1 year), or Arm C (doxorubicin, cyclophosphamide then weekly paclitaxel with a year of trastuzumab initiated concurrently with the paclitaxel). Patients received MUGA scans or echocardiography (ECHO) at baseline, 3, 6, and 9 months after registration, and after completion of chemotherapy. Cardiac events were defined as symptomatic CHF, definite CD (as a result of myocardial infarction, CHF, or arrhythmia), or probable CD (patient death without documented etiology). Three cardiologists independently investigated all CEs, and an event was confirmed if agreement was reached between at least two cardiologists. Of note, cardiac events were slightly higher in the group that received concurrent paclitaxel and trastuzumab (3.3% vs. 2.8%), and this group is most similar to Arm 2 in the NSABP B-31 trial. We also note here that the previously published GWAS analysis showed a decline in LVEF combined Arms B and C.

### 2.2 DNA samples

A total of 1,228 DNA samples were received from the NSABP Foundation, of which clinical data including baseline and follow-up MUGA scans and cardiac event status were available for 1,202 patients. Histograms indicating the distribution of age, the maximum decline in LVEF, and use of hypertensive medications in each treatement group for the 1,202 patients with available DNA samples are shown in **Supplementary Figure 1**.

### 2.3 Single-nucleotide polymorphism genotyping

Seven SNPs were genotyped using the Agena Bioscience platform. These SNPs included the most significantly associated SNPs with a maximum decline in LVEF in patients treated, reported in the initial GWAS of the N9831 clinical trial at *p* < 1 × 10^−5^: *TRPC6* rs77679196, *BRINP1* rs62568637, *LDB2* rs55756123, *RAB22A* rs707557, intergenic 6p22.3 rs4305714, *LINC01060* rs7698718 (7), and the *CBR3* rs1056892 (V244M) polymorphism that was associated with anthracycline-related heart failure in the literature (9–14) and significant at *p* < 0.01 in the N9831 GWAS.

### 2.4 Genotyping quality control

A total of 1,228 available DNA samples from the NSABP B-31 patients were plated into 14 × 96-well plates. Each 96-well plate included one blank well used for negative controls, and for each 96-well plate, two DNA samples were re-genotyped. Genotyping was 100% concordant for 28 duplicate DNA samples across 11 SNPs (308 genotypes), and all variants were within Hardy–Weinberg equilibrium (*p* > 0.05).

### 2.5 Statistical analysis

#### 2.5.1 Power analysis

All available DNA samples were requested from NSABP. For the purposes of power calculations, we assumed that the analysis would include *N* = 1,000 as approximately 20% of the requested samples would be excluded to limit the possibility of confounding due to genetic heterogeneity. From a clinical perspective, those variants with effects on cardiotoxicity that are of a magnitude sufficient to warrant consideration in making treatment decisions are of clinical relevance and are most important to replicate. However, even variants with relatively small effects (if real) are relevant in the larger context of seeking to understand the underlying mechanisms of cardiotoxicity. For linear regression of maximum decline in LVEF, we calculated power to detect effects of 1%, 2%, and 3% on the LVEF change with each copy of the risk allele and based on a one-sided test as replication would only be declared if the estimated effect size is in the same direction as in the discovery sample. For each scenario, power was estimated from results of 10,000 simulations of data and fitting of linear regression models with the following assumptions: (i) genotype frequencies follow the Hardy–Weinberg equilibrium; (ii) additive effect, residual standard deviation in LVEF changes of 8%. The assumption of 8% was based on a sample standard deviation of 7.6 having been obtained in the N9831 trial LVEF data. Simulations were conducted in the statistical programming language R version 3.2.3. These analyses suggested reasonable power (>80%) to detect effect sizes of magnitudes matching those estimated in the N9831 discovery analysis (**Supplementary Table 1**).

#### 2.5.2 Association analyses

The association of each SNP was tested with cardiac events using logistic regression (follow-up of 7 years as per (7)), and with the maximum decline in LVEF (up to 2 years from baseline to match analysis in N9831 GWAS) using linear regression, adjusting for age, baseline LVEF, and hypertension medication. All analyses were performed in plink 1.07 under an additive model. For the NSABP B-31 DNA specimens, GWAS data were not available to perform principal component analysis (PCA) of reported ethnicity against SNP genotypes as was done for N9831, where PCA1 and PCA2 were included as covariates. Therefore, to control for population differences and make comparable to the reported N9831 data, primary analyses of NSABP B-31 were performed in those patients who self-reported as white and non-Hispanic (*N* = 993). To test for the association of trastuzumab-related cardiotoxicity (quantitative analysis of decline in LVEF and logistic analysis of symptomatic CHF and CD), we tested the association of those patients who received chemotherapy and trastuzumab. To test for association of genetic variants with doxorubicin, we tested those patients who received only chemotherapy (no trastuzumab) and we also performed a combined analysis of NSABP B-31 patients, irrespective of treatment arm (as all patients received doxorubicin). Correction for multiple testing was not applied.

### 2.6 *In silico* analysis

The transcription factor binding affinity and the fold difference in binding between the two DNA sequences for each of the variants—*TRPC6* rs77679196, *BRINP1* rs62568637, *LDB2* rs55756123, *RAB22A* rs707557, intergenic 6p22.3 rs4305714, and intergenic 4q35.2 rs7698718—were predicted *in silico* using the Transcription factor Affinity Prediction (TRAP) web tools (15), sTRAP difference between two sequences with matrix file jaspar vertebrates, background model human promoters, and multiple test correction Benjamini–Hochberg.

## 3 Results

### 3.1 Associations of *TRPC6*, *BRINP1*, and *CBR3* with cardiac events in NSABP B-31 patients treated with chemotherapy plus trastuzumab

The GWAS subanalysis of the N9831 used a quantitative analysis of the maximum decline in LVEF in those patients treated with chemotherapy plus trastuzumab, and reported the genotype counts of the top loci (*p* < 1 × 10^−5^) in the subset of patients who experienced a cardiac event. Logistic regression was not performed in the initial publication due to the very small number of patients with cardiac events (*N* = 10). For the purpose of comparison with NSABP B-31, we now show these data side-by-side in **Tables 1A, B**. In the N9831 patients receiving chemotherapy plus trastuzumab, there were three loci (*LDB2*, *BRINP1*, and *TRPC6*) for which the frequency of minor allele was relatively low, <2%. In the N9831 patients, for each of these loci, we observed a higher frequency of the rare allele in patients who experienced cardiac events (**Table 1A**). In the NSABP B-31 patients, DNA samples were available from 19 patients experiencing a cardiac event. None of the three low-frequency variants were statistically significant (**Table 1B**). However, for *BRINP1* and *TRPC6*, the allele frequencies and odds ratios were in the same direction as observed for N9831 and we suggest that the reason for not attaining statistical significance (*p* < 0.05) in this case could also be a lack of statistical power (small number of cardiac events in combination with low allele frequency) rather than the null hypothesis being true.

**Table 1:** Genetic associations of cardiac events in NCCTG N9831 and NSABP B-31 patients treated with chemotherapy plus trastuzumab.

Table 1			A. N9831 chemotherapy + trastuzumab				B. NSABP B-31 chemotherapy + trastuzumab				C. NSABP B-31 chemotherapy only				D. NSABP B-31 chemotherapy and chemotherapy + trastuzumab combined			
CHR	*GENE/SNP*	A1	FA, *N* = 10	FU, *N* = 790	OR (CI)	*p*-value	FA, *N* = 19	FU, *N* = 485	OR (CI)	*p*-value	FA, *N* = 10	FU, *N* = 479	OR	*p*-value	FA, *N* = 29	FU, *N* = 964	OR	*p*-value
4	*LDB2* *rs55756123*	A	0.15	0.02	10.8 (2.79–41.8)	**0.0006**	0.00	0.01	0.00 (0–inf)	0.998	0.05	0.03	1.93 (0.25–14.7)	0.525	0.02	0.02	1.07 (0.14–7.96)	0.949
4	*LINC01060* *rs7698718*	A	0.20	0.22	0.86 (0.28–2.64)	0.793	0.36	0.21	2.15 (1.02–4.53)	**0.044**	0.19	0.20	0.92 (0.27–3.16)	0.897	0.31	0.21	1.66 (0.91–3.03)	0.098
6	*Intergenic* *rs4305714*	T	0.25	0.24	1.12 (0.39–3.23)	0.830	0.21	0.22	1.01 (0.43–2.34)	0.987	0.35	0.23	1.85 (0.69–4.99)	0.224	0.26	0.22	1.25 (0.66–2.35)	0.491
9	*BRINP1* *rs62568637*	A	0.05	0.01	7.78 (0.86–70.1)	0.067	0.03	0.02	1.75 (0.21–14.4)	0.604	0.00	0.02	0.00 (0–inf)	0.998	0.02	0.02	1.10 (0.14–8.50)	0.927
11	*TRPC6* *rs77679196*	A	0.10	0.01	12.97 (2.26–74.6)	**0.004**	0.03	0.01	2.29 (0.25–20.9)	0.463	0.05	0.00	12.84 (1.24–133.2)	**0.032**	0.03	0.01	4.67 (0.95–23.0)	0.058
20	*RAB22A* *rs707557*	T	0.25	0.40	0.53 (0.20–1.40)	0.199	0.45	0.36	1.50 (0.76–2.96)	0.240	0.45	0.37	1.28 (0.51–3.12)	0.598	0.45	0.37	1.37 (0.80–2.36)	0.252
21	*CBR3* *rs1056892*	A	0.65	0.64	1.11 (0.43–2.86)	0.835	0.75	0.62	2.13 (0.95–4.76)	0.067	0.75	0.62	1.82 (0.65–5.26)	0.259	0.75	0.62	1.92 (1.03–3.57)	**0.040**

**Table 1** shows the results of logistic regression analyses of cardiac events of the top six loci and a missense variant at CBR3 that were previously associated with maximum decline in LVEF in 800 patients in the NCCTG-N9831 trial, compared side-by-side in the NCCTG-N9831 and NSABP B-31 clinical trials.

CHR, chromosome; SNP, single-nucleotide polymorphism; A1, allele 1; FA, frequency of allele 1 in patients affected by cardiac events; FU, frequency of allele 1 in patients unaffected by cardiac events; OR, odds ratio; CI, upper and lower 95% confidence intervals. Bold values are p-values <0.05.

Among the more common variants, *CBR3* rs1056892 showed a trend for association in the NSABP B-31 patients, *p* = 0.067 (**Table 1B**), in the same direction as in other published studies (9, 11, 16–18). *LINC01060* rs7698718 was significantly more frequent in NSABP B-31 patients who experienced a cardiac event, cases allele A = 0.36, controls allele A = 0.21, *p* = 0.044 (**Table 1B**). These data are in the opposite direction to that observed in the N9831 patients, for which we did not observe a significant difference in allele frequency in patients with cardiac events. Furthermore, in the quantitative analysis of the maximum decline in LVEF in the N9831 patients, for the same variant, there was no association in the NSABP B-31 patients in either direction (**Table 2**).

**Table 2:** Linear regression of genetic variants with the maximum decline in LVEF in NCCTG N9831 and NSABP B-31 patients treated with chemotherapy plus trastuzumab.

				N9831 GWAS, *N* = 800		NSABP B-31 replication, *N* = 504	
CHR	GENE	SNP	A1	Beta (95% CI)	*p*-value	Beta (95% CI)	*p*-value
4	*LDB2*	rs55756123	A	−6.11 (−8.32 to −3.89)	8.93E−08	1.76 (−3.25 to 6.77)	0.491
4	LINC01060	rs7698718	A	1.78 (1.01 to 2.56)	7.73E−06	0.09 (−1.09 to 1.29)	0.879
6	intergenic	rs4305714	T	−1.87 (−2.63 to −1.12)	1.39E−06	−0.21 (−1.37 to 0.96)	0.728
9	*BRINP1*	rs62568637	A	−8.50 (−11.81 to −5.19)	6.01E−07	3.83 (0.17 to 7.50)	0.041
11	*TRPC6*	rs77679196	A	−7.00 (−10.05 to −3.96)	7.72E−06	−0.47 (−5.81 to 4.86)	0.862
20	*RAB22A*	rs707557	T	1.46 (0.83 to 2.08)	5.62E−06	−0.01 (−1.01 to 0.99)	0.984
21	*CBR3*	rs1056892	A	1.04 (0.35 to 1.72)	0.003	−0.01 (−1.05 to 1.02)	0.980

**Table 2** shows the results of linear regression analyses of the top six loci and a missense variant at CBR3 that were previously associated with maximum decline in LVEF in 800 patients in the NCCTG-N9831 trial, compared side-by-side in the NCCTG-N9831 and NSABP B-31 clinical trials. Beta is the max decline in LVEF for each copy of the risk allele. Abbreviations: CHR, chromosome; SNP, single-nucleotide polymorphism; A1, allele 1; CI, upper and lower 95% confidence intervals.

### 3.2 *TRPC6* rs77679196 and *CBR3* V244M are associated with cardiac events in NSABP B-31 patients treated with chemotherapy

In the N9831 patients, the minor alleles at *LDB2* rs55756123 and *TRPC6* rs77679196 were significantly associated with cardiac events, OR 10.8, *p* = 0.0006 and OR 12.97, *p* = 0.004, respectively, but it was not clear if the association was the result of chemotherapy, trastuzumab, or both. Therefore, we next looked at patients who were only treated with chemotherapy (**Table 1C**) and in both treatment arms combined (**Table 1D**) with the hypothesis that the observed cardiac events were the result of doxorubicin rather than trastuzumab (all patients in both trials received doxorubicin). In the NSABP B-31 patients, no association was observed with *LDB2* rs55756123, in the chemotherapy only group or in the combined analysis.

*TRPC6* rs77679196 was significantly associated with cardiac events in NSABP B-31 patients treated with chemotherapy only, OR 12.84, *p* = 0.032 (**Table 1C**), in which the minor allele was in excess in patients with cardiac events (0.05 in cases vs. 0.00 in controls), the same direction observed in N9831.

*In silico* analysis was performed to predict different transcription factor binding for each risk variant, the most significant findings of which were for *TRPC6* rs77679196. For this variant, it was predicted that the associated allele (A) significantly reduces the binding of the inhibitory transcription factor, REST, by ~3-fold, *p* = 8.27 × 10^−7^, which would potentially increase *TRPC6* expression. In animal and human studies, increased TRPC6 expression is associated with pathologic cardiac remodeling (19), and in our own studies of N9831, imputed *TRPC6* expression in tissue from left ventricle was associated with decline in LVEF (20).

*CBR3* rs1056892, V244M, was associated with a decline in LVEF in the initial N9831 GWAS analysis but was not associated in the binary analysis of N9831 cardiac events (*N* = 10). In the NSABP B-31 patients treated with chemotherapy only, the V244 allele was more frequent in patients who experienced cardiac events (0.75 in cases vs. 0.62 in controls) but was not statistically significant, OR 1.82, *p* = 0.259 (**Table 1C**). In the larger combined analysis of patients treated with either chemotherapy or chemotherapy plus trastuzumab, where 29 patients experienced a cardiac event, the major G allele, V244, was significantly more frequent (cases G = 0.75, controls G = 0.62), OR= 1.92, *p* = 0.04 (**Table 1D**).

### 3.3 Genetic associations of the maximum decline in LVEF were uninformative in the NSABP B-31 patients treated with chemotherapy plus trastuzumab

GWAS primary analysis of patients in the N9831 clinical trial (7) used a linear regression of the maximum decline in LVEF with age, baseline LVEF, and hypertensive medications as covariates, in the patients who received chemotherapy (doxorubicin and paclitaxel) plus trastuzumab. Using the same analysis in NSABP B-31 patients treated with chemotherapy plus trastuzumab, we did observe an association at *BRINP* rs62568637, *p* = 0.041 (**Table 2**), but as shown in the box plot (**Supplementary Figure 2D**), the median change in LVEF was higher in patients with one copy of allele A relative to patients with 0 copies of allele A, whereas in the N9831 patients, the median change in LVEF in patients was lower in patients with one copy of allele A relative to patients with 0 copies of allele A. Therefore, none of the associations with the top six GWAS loci, or *CBR3* rs1056892, V244M were associated in the same direction as observed in N9831 at *p* < 0.05 (**Table 2**).

## 4 Discussion

In this study, we assessed genetic variants that were previously associated with cardiotoxicity in the NCCTG N9831 trial for replication in the NSABP B-31 trial. Pharmacogenetic studies of cardiotoxicity in cancer patients have proved challenging due to the use of multiple cardiotoxic therapies (likely with different mechanisms of toxicity) in each patient regimen, different definitions of cardiotoxicity, variability in echocardiography measurements, relative low frequency of therapy-related CHF, lack of control for known risk factors, and a lack of control for population stratification (an uneven distribution of allele frequencies in affected and unaffected patient groups due to differences in ancestry rather than true association with disease).

The current study was able to address some of these problems by side-by-side analysis of the NCCTG N9831 and NSABP B-31 clinical trials using the same chemotherapy and trastuzumab regimen in patients with high-risk early HER2+ breast cancer. Both trials used a control arm of chemotherapy only, which was tested against chemotherapy plus trastuzumab, and both trials used baseline and serial LVEF measurements and followed cardiac events out to a median of >7 years, allowing for testing of genetic associations resulting from chemotherapy (likely doxorubicin) and from chemotherapy plus trastuzumab. Two loci, *TRPC6* and *CBR3*, were associated with cardiac events in the NSABP B-31 patients and analyses suggest that both associations are the effects of chemotherapy rather than trastuzumab and further extend the evidence for both as putative risk genes for doxorubicin-induced heart failure.

Firstly, *TRPC6* is a non-selective calcium channel known to be involved in heart failure in general and in cardiac remodeling in animal models (19), and in our previous studies, *TRPC6* knockout (21) and pharmacological inhibition of *TRPC6 in vitro* and in wild-type mice (20) attenuated doxorubicin-induced cardiotoxicity, respectively. Furthermore, a study by Kuwahara et al. showed that TRPC6 was upregulated in mouse hearts subjected to pressure overload and in failing human hearts (19) and transcriptome-wide analysis (TWAS) of the GTEX tissues from heart left ventricle and decline in LVEF in the N9831 GWAS dataset demonstrated an association with *TRPC6* (20). Specifically, relating to the risk variant, rs77679196 is located in *TRPC6* intron 1, and lies within a transcription factor binding site for the transcriptional repressor REST (RE1-silencing transcription factor). REST is associated with cardiac development and maintenance of normal cardiac function in adult hearts (22–25), and inhibition of REST in the heart has been shown to lead to cardiac dysfunction accompanied by re-expression of various fetal genes, including those encoding fetal ion channels (24). Our *in silico* analysis of the rs77679196 is also in line with this finding, in that the associated allele has significantly reduced binding of REST to *TRPC6*, suggesting higher TRPC6 in individuals with the putative risk allele. Therefore, taken together, our data and other published studies suggest that (1) higher expression levels of *TRPC6* are associated with cardiac remodeling and heart failure; (2) genetic variants at *TRPC6* are associated with *TRPC6* expression levels; and (3) inhibition of *TRPC6* may be a useful cardioprotective therapy for doxorubicin-induced heart failure.

Secondly, we observed the association of *CBR3* (Carbonyl-reductase 3) V244M, a polymorphism that has previously been associated with anthracycline-induced heart failure in childhood cancer patients (9, 16, 17) and in patients with breast cancer (7, 10, 11, 18). The major allele variant, V244 (allele G), is associated with cardiotoxicity, and in biochemical assays, the same allele metabolizes doxorubicin 2.6 times faster than M244 (allele A) (9, 26), and in line with these existing studies, in the NSABP B-31 patients, V244 (allele G) was more frequent in patients experiencing a cardiac event.

A major goal of this study was to identify genetic variants associated with trastuzumab-related cardiotoxicity, either as a decline in LVEF or with cardiac events in a dataset from a clinical trial. Two common genetic polymorphisms, Ile655Val and Pro1170Ala in the trastuzumab target, *ERBB2*, have previously been associated with increased risk of trastuzumab-related cardiotoxicity in retrospective studies (27–31), but sample sizes were small, chemotherapy regimens and doses were heterogeneous, and analyses did not incorporate known risk factors such as age and baseline LVEF at the time of treatment. As previously described, and under multiple definitions of cardiotoxicity and linear and logistic models, we did not observe any association of these variants in patients in the N9831 clinical trial (7). Ile655Val and Pro1170Ala were not included in the current study because they were previously tested for association with cardiotoxicity by the NSABP group, and similarly to that observed in the N9831 patients, in the NSABP patients, there were no associations with cardiotoxicity (personal communication with Dr. Kay Pogue-Geile, NSABP).

## 5 Limitations

There are several limitations to this study. Firstly, the small number of cardiac events (***N*** = 29). Therefore, the lack of statistical significance for some SNPs may reflect low statistical power rather than true lack of association, and therefore, the possibility of a type II error (i.e., a false-negative finding) is important to consider. This is particularly a concern when drawing conclusions about subgroups in which the statistical power may be lower for some analyses due to the reduced sample size. Secondly, the definition of cardiac events did not include serum biomarkers or strain parameters that are more widely available today as these trials were completed over 15 years ago. Thirdly, these were phase III clinical trials, which gives the advantage of larger numbers of patients, but adds the disadvantage of echocardiograms being completed at multiple clinical sites with different operators, thus increasing variability in LVEF measurement. Fourthly, using a linear approach of the maximum decline in LVEF as opposed to a binary analysis of decline in LVEF >10 points may be less clinically relevant, although we emphasize that the goal of this study is to replicate previous findings of the genes that may be involved in cardiotoxicity. Finally, three SNPs were of relatively low frequency (MAF <2%), which, combined with the small number of cardiac events, would lower statistical power, even with the large odds ratios reported for the same SNPs in N9831 and regardless of whether the method of analysis used a linear approach of maximum decline in LVEF or a binary approach of cardiac event or decline in LVEF >10 points.

## 6 Conclusions

These new data replicated the previous genetic association of *TRPC6* with doxorubicin-induced cardiac events. Association with the *CBR3* V244M variant was significant in the group of NSABP B-31 patients treated with doxorubicin plus trastuzumab. We conclude that this association is the effect of doxorubicin and is observed due to the larger sample size rather than the effect of trastuzumab. These results generate the hypothesis that *TRPC6* and *CBR3* are risk genes for doxorubicin-related cardiotoxicity for which additional fine mapping of the associated genetic variants and more detailed clinical data are required to improve clinical relevance.

## Data availability statement

The data analyzed in this study is subject to the following restrictions: Sharing of individual level genotype or individual clinical data is not permitted under a sharing agreement with the NSABP Foundation. Requests to access these datasets should be directed to the corresponding author and the NSABP Foundation.

## Ethics statement

The studies involving human participants were reviewed and approved by Mayo Clinic Institutional Review Board. The ethics committee waived the requirement of written informed consent for participation.

## Author contributions

NN, PA, JR, JH, KR, GC-O, and EP designed and conducted the study and analyses of the data. NN performed genotyping and statistical analysis and wrote the manuscript. ECC provided statistical support. GY and RC provided NSABP/NRG clinical data. KP-G provided DNA samples. CL and HF processed blood specimens and plated DNA samples for shipping. PR, EM, SS, CG, and NW were the clinical trialists for NSABP B-31. NW is the NSABP Chairman. SP was the Director of NSABP Pathology and B-31 pathologist. All authors critically reviewed the manuscript. All authors contributed to the article and approved the submitted version.
