# Supplementary material for: Replication of genetic associations of chemotherapy-related cardiotoxicity in the adjuvant NSABP B-31 clinical trial
Source: Front Oncol. 2023 May 25;13:1139347. doi: 10.3389/fonc.2023.1139347 (PMC10248403; doi:10.3389/fonc.2023.1139347)
Supplement: Supplementary file 1 [file DataSheet_1.docx]

**Supplementary Figure 1. Distribution of maximum decline in LVEF, age and use of hypertensive medication at the time of randomization to treatment arms of chemotherapy or chemotherapy plus trastuzumab in 1,202 patients with available DNA samples from the NSABP B-31 clinical trial.**


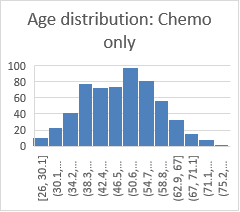

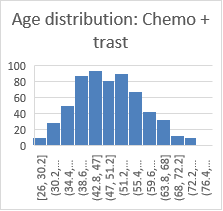


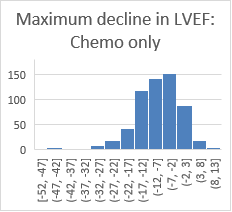

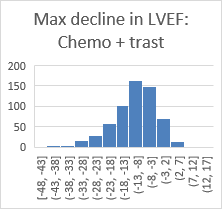


**Supplementary Figure 2. Maximum decline in LVEF by risk allele in patients treated with chemotherapy plus trastuzumab.** The top associated loci of maximum decline in LVEF, p<1x10^-5^ (A-F) from previous GWAS analysis of N9831 were not associated in the same direction, p<0.05 in NSABP B-31. In this analysis, *BRINP1* rs62568637 (D) was associated with maximum decline of LVEF in the NSABP B-31 patients, but in the opposite direction to that observed in N9831.

**Supplementary Table 1. Initial power calculations for linear regression analysis of maximum decline in LVEF, assuming N=1000.**

| **MAF** | **Power to detect given effect on LVEF reduction with each copy of minor allele (1-sided p=0.0250)** | | | | **Effect sizes for given MAF or lower estimated in N9831** |
| --- | --- | --- | --- | --- | --- |
|  | **1%** | **2%** | **3%** | **5%** |  |
| 0.01 | 10 | 22 | 38 | 78 | ≥ 6.2 |
| 0.02 | 14 | 33 | 65 | 96 | ≥ 4.7 |
| 0.05 | 21 | 69 | 96 | >99 | ≥ 3.1 |
| 0.10 | 38 | 92 | >99 | >99 | ≥ 3.1 |
| 0.20 | 63 | 99 | >99 | >99 | ≥ 3.1 |
| 0.50 | 80 | >99 | >99 | >99 | ≥ 1.3 |

MAF, minor allele frequency
